# ExtRanFS: An Automated Lung Cancer Malignancy Detection System Using Extremely Randomized Feature Selector

**DOI:** 10.3390/diagnostics13132206

**Published:** 2023-06-29

**Authors:** Nitha V. R., Vinod Chandra S. S.

**Affiliations:** Department of Computer Science, University of Kerala, Thiruvananthapuram 695581, India; vinod@keralauniversity.ac.in

**Keywords:** lung cancer malignancy detection, transfer learning, feature extraction, feature selection, extratree classifier, deep learning

## Abstract

Lung cancer is an abnormality where the body’s cells multiply uncontrollably. The disease can be deadly if not detected in the initial stage. To address this issue, an automated lung cancer malignancy detection (ExtRanFS) framework is developed using transfer learning. We used the IQ-OTH/NCCD dataset gathered from the Iraq Hospital in 2019, encompassing CT scans of patients suffering from various lung cancers and healthy subjects. The annotated dataset consists of CT slices from 110 patients, of which 40 were diagnosed with malignant tumors and 15 with benign tumors. Fifty-five patients were determined to be in good health. All CT images are in DICOM format with a 1mm slice thickness, consisting of 80 to 200 slices at various sides and angles. The proposed system utilized a convolution-based pre-trained VGG16 model as the feature extractor and an Extremely Randomized Tree Classifier as the feature selector. The selected features are fed to the Multi-Layer Perceptron (MLP) Classifier for detecting whether the lung cancer is benign, malignant, or normal. The accuracy, sensitivity, and F1-Score of the proposed framework are 99.09%, 98.33%, and 98.33%, respectively. To evaluate the proposed model, a comparison is performed with other pre-trained models as feature extractors and also with the existing state-of-the-art methodologies as classifiers. From the experimental results, it is evident that the proposed framework outperformed other existing methodologies. This work would be beneficial to both the practitioners and the patients in identifying whether the tumor is benign, malignant, or normal.

## 1. Introduction

**Figure 1:**
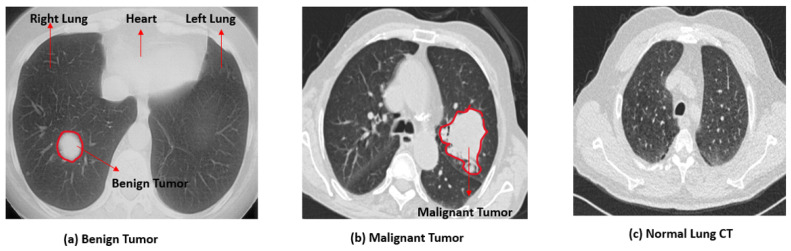
Annotation of malignancy in lung tumor.

Lung cancer is considered the second most common cancer worldwide, and it usually begins in the lungs and spreads to nearby tissues or fluids easily. Normal cell division and growth are adequate for repairing the body’s cells. Still, when the growth is abnormal and uncontrollable, tumors tend to develop. Lung tumors may not have specific symptoms initially and are broadly divided into benign (non-cancerous) and malignant (cancerous). When a patient is diagnosed with a lung tumor, the next step is to identify whether the tumor is benign or malignant. Benign tumors are non-cancerous masses that grow slowly. The cells within the benign tumor always lie within the tumor boundary and will not spread or invade nearby tissues. Sometimes benign tumors can evolve into enormous masses of tissues with well-defined boundaries and are not harmful. However, malignant tumors are cancerous tumors that can spread to any body part through the circulatory system or the lymph. These tumors invade nearby cells easily and will not have well-defined boundaries. Owing to the rapid proliferation, these malignant tumors may eventually return even after surgery. The differentiation of benign and malignant tumors is clearly illustrated in the annotated Figure 1. Figure 1a is an annotated benign tumor, Figure 1b represents a lung CT with a malignant tumor, and Figure 1c is a normal lung CT image. There are various imaging modalities for lung cancer detection. Computed Tomography (CT), PET (Positron Emission Tomography), and MRI (Magnetic Resonance Imaging) are widely used for lung abnormality detection.

Sometimes even an experienced physician may miss a lung tumor because of an error in observation, low image quality, or wrong patient position. At times, the size of the cancer tissue and the location can also lead to a misdiagnosis. Hence, Computer Aided Detection (CAD) can be effectively utilized to lessen manual and technical errors in diagnosis and can be employed for the early detection of lung cancer. Artificial intelligence is a broad domain with unlimited applications, including clinical decision-making [1]. Various Machine Learning and Deep Learning algorithms can effectively automate disease or abnormality detection, localization, and quantification [2,3]. Initially Niki et al. [4] experimented with lung CT images to detect whether the tumor was cancerous in 2001 by utilizing CAD techniques, whereas Aberle et al. [5] analyzed how low-contrast CT images can be used in lung cancer diagnosis and thereby reducing mortality. Awai et al. [6], and Mozaffary et al. [7] utilized CAD for identifying small lung nodules from computed tomography images. Sahiner et al. [8] identified that CAD has better performance when compared to radiologists’ visualization and can eliminate manual misdiagnosis errors too.

As part of disease diagnosis, various pre-processing techniques can be applied to medical imaging. Studies reveal that pre-processing enhances the efficiency of the model. Researchers proposed various methodologies for lung cancer diagnosis using machine learning techniques. In the case of machine learning, relevant features must be manually extracted with the help of domain expertise, but sometimes inaccurate feature selection may impact the classifier. Chaganti et al. [9] suggested that in image classification, the traditional machine learning approach is superseded by deep learning because of its computational efficiency. Deep learning enables the extraction of fundamental and complicated features automatically rather than manual feature extraction. Deep learning enables us to quantify the tumor size, thereby measuring the tumor size and depth. Our proposed method employs lung CT imaging modality for malignancy detection.

In our proposed model, we choose transfer learning as a feature extractor which enables us to utilize the information learned from one dataset to another and works well even with inadequate datasets. We have utilized pre-trained VGG16 as a feature extractor by freezing all convolution layers. To reduce the computational complexity, an extremely Randomized Tree Classifier is employed as a feature selector and is fed to the Multi-Layer Perceptron (MLP) Classifier for classification. The model could improve performance when compared to other machine learning techniques.

### Related Works

The advancements in artificial intelligence and machine learning can be used in various stages of lung cancer. When a patient has no symptoms, machine learning can be used for the prediction and risk assessment of lung cancer based on multiple factors such as family history, smoking history, etc. If a patient is symptomatic, these techniques can be used for disease detection, localization, and segmentation. Another use case of machine learning is in assessing disease progression and treatment response prediction. Danjuma et al. [10] conducted a study on the life expectancy prediction of lung cancer patients by utilizing Naive Bayes, Decision Tree, and Artificial Neural Network algorithms. Zehra et al. [11] compared various machine learning algorithms such as SVM, KNN, and Logistic Regression and out of which the SVM Classifier achieved the best accuracy. Radhika et al. [12] employed a comparative study on various machine learning algorithms for lung cancer detection.

Many works were conducted, including pre-processing, contrast enhancement, and segmentation. Image pre-processing and contrast improvement are vital steps in digital image processing. Segmentation techniques enable one to separate the foreground from the background. In machine learning algorithms, manual feature extraction has to be performed for image classification. Tiwari et al. [13] suggested the mask3 FCM and TWEDLNN algorithms for lung cancer diagnosis. The work involves lung segmentation by utilizing OTSU thresholding, followed by contrast enhancement. Features are extracted from the thresholded images and are fed to the classifier, and the model achieved an accuracy of 96.00%. Surendar et al. [14] proposed a hybrid deep learning model utilizing image pre-processing with non-local means filter followed by segmentation using thresholding. The features are extracted from the segmented image by a grey-level matrix accompanied by feature selection with a binary grasshopper optimizer. The selected features are classified using a hybrid deep neural network model with a sine-cosine crow search algorithm.

The significant contributions of the work are as follows:

- Developed a framework for predicting lung cancer malignancy at an early stage. Sometimes even an expert radiologist may miss a relatively small lung tumor tissue which can be life-threatening.
- The implication of various tree splitting criteria in ExtraTreeClassifier as feature selector is compared.
- A comparative study is performed on various CNN models as feature extractors, with further consideration of the performance of the proposed framework with existing systems.
- A comparison is performed with other state-of-the-art machine learning classifiers.

## 2. Materials and Methods

**Figure 2:**
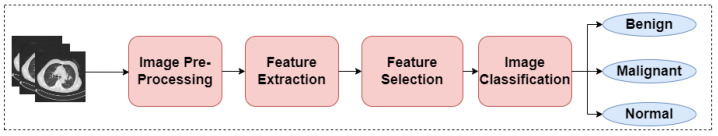
Pipeline of the proposed methodology.

The pipeline of the proposed framework is shown in Figure 2. We have used a publicly available IQ-OTH/NCCD dataset consisting of CT images of lung cancer patients with benign, malignant, and normal cases. Initially, the images are pre-processed, followed by extracting relevant features. To reduce the computational complexity, feature selection is performed using ‘ExtraTreeClassifier’. The selected features are fed as neurons to the input layer of the Multi-Layer-perceptron (MLP) Classifier for classifying tumors into either benign, malignant, or normal cases. The models were trained and tested in Python 3.8.10 on NVIDIA Tesla V100-PCIE Graphics Processing Units (GPU) deployed on a high-performance computing cluster with 1 Teraflop.

### 2.1. Dataset

We utilized the lung cancer dataset from the Iraq-Oncology Teaching Hospital/National Center for Cancer Diseases (IQ-OTH/NCCD) from Kaggle [15], which was published in 2019 and comprises CT scans of individuals who have been diagnosed with lung cancer. The collection includes images of 110 people with lung cancer in various stages, including benign, malignant, and normal. These 110 patients vary in gender, age, education, etc. Out of these, 40 patients were diagnosed with a malignancy in lung tumors, 15 patients had lung tumors that were not cancerous (benign), and 55 patients had normal lungs. Each patient has roughly 80–200 slices of a CT scan, each with a 1mm slice thickness. These patients come in various ages and genders; they do not all belong to the same category. The dataset comprises 1097 CT images of two different lung cancer types and a normal category. The first category is benign lung cancer containing 120 CT images, and the second category includes 561 malignant lung CT images. The third category is normal lung CT containing 416 images. Tumors are typically classified into either benign or malignant. Benign tumors are non-cancerous, whereas malignant tumors are cancerous. Here, 80% of the data are used for training, and 20% of the data are used for testing.

### 2.2. Proposed Methodology

**Figure 3:**
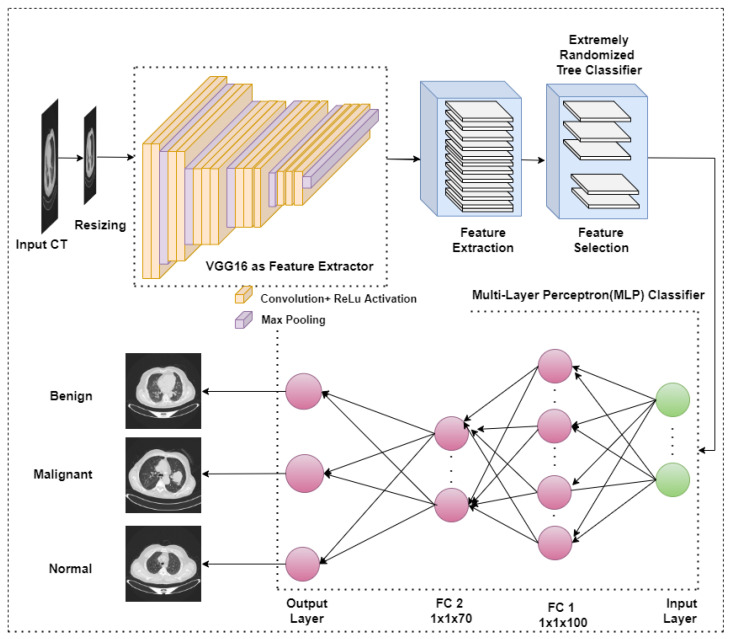
Architecture of ExtRanFS framework.

This paper proposes a Deep Transfer learning Based Lung Cancer Detection System consisting of three tasks such as feature extraction, feature selection, and classification. The detailed architecture of the proposed methodology is illustrated in Figure 3. Initially, the Lung CT images are acquired from the ‘IQ-OTH/NCCD’ dataset. Let *I_Ori_* be the initial image which is resized to 224 × 224 × 3 dimension and is represented by *I_Rsize_* as in Equation (1). Keras provides the VGG16 model pre-trained on the huge ImageNet dataset.

(1) $$I_{Rsize}=Resize\left(I_{\mathrm{Ori}}\right)$$

The resized images are inputted into the VGG16 model, with pre-trained weights on ImageNet Dataset.

The model has five blocks of 3 × 3 convolution layers with five max-pooling layers in between. The feature extraction is performed by the convolution and pooling layers of VGG16 by leaving behind the fully connected layer. Hence the Include_Top is set as False to exclude the final classification layer of VGG16. The pre-trained model outputs a three-dimensional feature stack after feature extraction.

An extremely randomized tree classifier is applied on the feature stack as a feature selector. As a result of feature selection, the training and testing of the classifier take less computational time. The model performs better because feature selection reduces execution time, eliminates duplicate and irrelevant features, and removes misleading characteristics. The model training becomes faster with fewer features, which eliminates overfitting. The model becomes less intricate, more economical, and more generic. The features selected by the extremely randomized tree classifier are given to the input layer of the Multi-Layer Perceptron (MLP) Classifier. The classifier has two fully connected layers with 100 and 70 neurons, respectively, with ReLu activation. The last output layer consists of three neurons with Softmax activation. The model is trained using the selected features, and the model predicts the testing data efficiently. The overview of ExtRanFS framework is shown in Algorithm 1.
**Algorithm 1** ExtRanFS: Proposed Framework  **Input**: Lung CT Image (I_Ori_)  **Output**: Classified Lung CT Image (I_Out_)1:**procedure** PROC12:    *Read each (I_Ori_) in the Dataset*3:    $\mathrm{Resize}\;(I_{\mathrm{Ori}})\;\mathrm{into}$ *(224,224,3)*4:    $\mathrm{Extract}\;\mathrm{Features}\;\mathrm{from}\;(I_{\mathrm{Ori}})\;\mathrm{Using}$ *Pre-trained VGG16 to F*5:**end procedure**6:**procedure** PROC27:      Fs←*Select the Best Features using ExtraTreeClassifier*8:      *Apply Label Encoding*9:      *Divide the Dataset into Training and Testing*10:    *Input Selected Features to Multi-Layer Perceptron (MLP) Classifier*11:    *Predict the lung cancer malignancy into the classes benign, malignant, or normal*12:**end procedure**

#### 2.2.1. VGG16 as Feature Extractor

The proposed methodology is implemented in a Python environment using the IQ-OTHNCCD dataset. Transfer learning is a strategy that allows the knowledge gained in one task to be applied to another, improving the performance of the second work. Transfer learning eliminates the need for an extensive dataset and training the model from scratch. A transfer learning approach has a lot to offer. When compared to a transfer-learned model, normal convolutional neural networks require days to train. Since the transfer learned model was trained on a huge dataset, it outperforms the model that was created from scratch and can already recognize key features with less training data. In our case, we have an inadequate dataset with three classes: benign, malignant, and normal. To deal with the insufficient dataset, it is highly recommended to use the features identified by another model trained on a larger dataset. This technique is known as transfer learning, where the knowledge gained on one model is re-applied to another problem. Transfer learning can be used as a feature extractor or for fine-tuning. Here we used VGG16 for feature extraction only.

**Figure 4:**
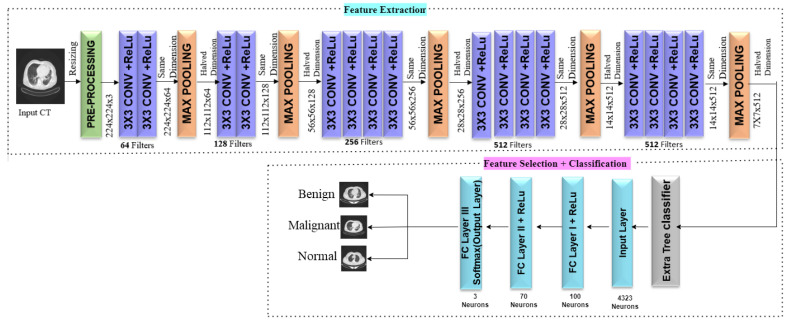
Detailed architecture of the proposed framework.

Filters are used in the convolution layer to extract features from images. The kernel size and stride are the two most crucial variables. The pooling layer’s purpose is to lower the network’s spatial size so that there are fewer parameters and computations to be made. The fully connected layers in pre-trained models are replaced with the classifier as per our requirement. The VGG16, a type of convolutional neural network, was unveiled as a part of the ILSVRC competition. It has 16 layers and is trained on an ImageNet dataset with 1000 classes. The detailed architecture of the proposed framework is illustrated in Figure 4. Initially, we imported VGG16 from keras.applications with an input image size. The input images in the dataset are in the dimension of 512 × 512 × 3 RGB images. To use the dataset for the VGG16 model, the images are resized to 224 × 224 × 3. The Include = Top is set to False, which signifies omitting the topmost classification layer, and the weights are imported from ImageNet classification from which VGG16 is pre-trained. We have used VGG16 only for feature extraction, which contains five blocks of convolution layers. The first convolution block consists of two 3 × 3 Convolution layers with 64 filters, stride 1, and ReLu activation. Each convolution block is followed by a Max-pooling layer with a 2 × 2 window and stride 2. The second convolution block has two 3 × 3 Convolution layers with 128 filters, stride 1, and ReLu activation. The third block of convolution has three 3 × 3 Convolution layers with 256 filters, stride 1, and ReLu activation. The fourth and fifth blocks of convolution have three 3 × 3 Convolution layers, 512 filters, stride 1, and ReLu activation.

**Figure 5:**
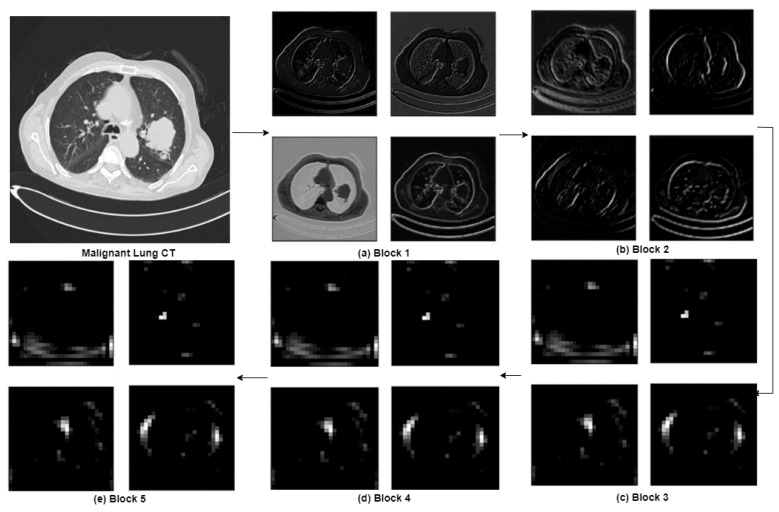
Visualization of the feature maps extracted from each block in the VGG16 Model.

The detailed illustration of feature maps generated at each convolution block is projected as Figure 5. The pre-trained model’s convolution and max-pooling layers gather features from the image and output a 3-Dimensional feature stack. The first five convolution layers of the pre-trained VGG16 extracted visual features necessary for classification. From this, it is evident that the initial layers extract more prominent and finer details when compared to the last convolution layers. From the initial convolution layers to the final layers, the model can abstract the image details into broader ideas to lead to categorizing the image. Before utilizing these features in machine learning classifiers, they will be flattened to 1-Dimension. The extracted flattened features are given to the Extremely Randomized Classifier for feature selection. The original VGG16 architecture has three fully connected layers with 4096 neurons at two dense layers and 1000 neurons in the output layer. The selected features are fed to the input layer of the MLP Classifier with 4323 neurons. We removed the fully connected classifier of VGG16, and instead of the last three fully connected layers (classifier part) of the VGG16 model, we used an MLP Classifier with two dense layers as fully connected layers with 100 and 70 neurons, respectively. The output layer has a fully connected layer with three neurons with Softmax Activation.

#### 2.2.2. Extremely Randomized Ensemble Classifier as Feature Selector

An extremely Randomized Trees Classifier is an ensemble learning algorithm that resembles the Random Forest Classifier, also known as Extra Trees Classifier. This method accomplishes classification by aggregating the output of numerous decision trees that make up a forest. The decision trees in Extra Tree Classifier are constructed from the training data, and the splitting criteria are decided mainly by Gini Index or Entropy. Gini Index and Entropy are used for assessing the information gain and are considered as a measure of the impurity of a node. When splitting the node, if a node has multiple classes, the class is highly impure. Entropy is calculated using Equation (2), and Gini Index is calculated in Equation (2). *Pc_(i)_* is the probability of class *c_(i)_* in a node, and n is the number of classes.

(2) $$Entropy=\sum_{i=1}^{n}-P\left(c_{i}log_{2}\left(P\left(c_{i}\right)\right)\right)$$

(3) $$Gini=1-\sum_{i=1}^{n}P^{2}c\left(i\right)$$

If the Gini Index is 0, it means that when two classes are split, just one class shows on one side while the other class is represented on the other. This division is thought to be perfect and is considered a pure classification. The Gini Index calculation is simple when compared to the Entropy calculation. In Entropy, in addition to probability, the log of the probabilities has to be computed and hence will not be computationally efficient. The Extra Tree Classifier fits several extra trees on different samples of the dataset and utilizes averaging ensemble technique to increase the accuracy and thereby control overfitting. The ExtraTreeClassifier is an ensemble technique similar to the Random Forest. The bootstrapping technique is used in the Random Forest Classifier, and the sub-samples are created from the input using replacement. Instead of using bootstrapping, Extra Trees use the entire training sample, which minimizes bias. Random Forest employs an optimal split when dividing trees, whereas Extra Trees uses a randomized split. Random Forest and Extra Trees chose the best feature after deciding on the split point. Extra Trees, when compared to the Random Forest, adds randomness by selecting each node’s split point at random, which unquestionably lowers variance.

Extra Trees are faster than Random Forest. Hence this technique is more computationally efficient and takes less time to execute. The classifier employs averaging to improve the ensemble classifier accuracy and will also lessen over-fitting by fitting several randomized decision trees. We can adjust the number of trees and the tree-splitting criteria. The number of trees is generally in the range of 10 to 100, and as the number of trees increases, the performance improves, but obviously, the computation time will be more. It is obvious that beyond a certain no of trees, the results are not improving. Either Gini or Entropy can be used as the tree-splitting criterion. Entropy and the Gini Index are used to evaluate information gain and are seen as indicators of a node’s impurity. If a node contains several classes after splitting, the class is quite impure. When contrasted with the Entropy computation, the Gini Index calculation is simpler. Entropy is not computationally efficient since, in addition to probability, the log of the probabilities also needs to be computed.

#### 2.2.3. Classification

The features selected from the Extremely Randomized Tree Classifier are inputted into the Multi-Layer Perceptron(MLP) Classifier. The input is flattened in the sequential model to produce a single input-single output model. The input layer consists of 4323 neurons comprising the features selected by ExtraTreeClassifier. The next two dense layers make up the fully connected hidden layers, and the final output layer, which is the final dense layer, is accountable for deciding the image class. The classifier has two fully connected dense layers of 100 and 70 neurons, respectively. Each dense layer is activated by ReLu activation. The model is optimized using the Adam optimizer, and loss is calculated using sparse categorical cross-entropy. The last output layer had three neurons and was activated by Softmax activation. The Batch size is fixed at 32, and the number of epochs is 15. The proposed MLP Classifier classified the input image into either of the three classes with an accuracy of 99.09%.

#### 2.2.4. Evaluation

The assessment of the model is measured in terms of quality metrics such as accuracy, precision, sensitivity, specificity, and F1-Score. The performance of the classifier is evaluated using the confusion matrix. The accuracy and loss of the model, along with the Receiver Operating Characteristics (ROC) Curve, is also illustrated in Section 3.

## 3. Results and Discussion

We discuss the experiments conducted by various pre-trained models as feature extractors. The performance of the MLP Classifier is compared with other state-of-the-art methodologies as classifiers. On NVIDIA Tesla V100-PCIE Graphics Processing Units (GPU) set up on a high-performance computing cluster with 1 Teraflop, all models were trained and tested in Python 3.8.10.

### 3.1. Feature Extraction by VGG16

The proposed methodology is implemented in a Python environment using the IQ-OTHNCCD dataset. In this study, transfer learning is applied as a feature extractor to deal with insufficient datasets. We utilized VGG16 as a feature extractor by excluding the top classification layers. Figure 5 represents the visualization of feature maps extracted from each convolution block of the VGG16 feature extractor.

To compare the feature extraction with other state-of-the-art methodologies, the experiment was performed with different pre-trained models as feature extractors, such as InceptionV3, Xception, and MobileNetV2. Google unveiled the Inception V1 architecture, commonly known as GoogleNet, in 2015. Later, Inception-V2, a variation of Inception V1 with Batch normalization, was developed. The Inception-V3 with BN auxiliary is an improved version of the Inception-V2. The main design of Xception consists of three flows: entry, middle, and exit, each with Convolution and Separable Convolution block along with Residual connection. MobileNetV2 is another CNN developed by Google with residual skip connections between bottleneck layers. The results are discussed in Section 3.3.

### 3.2. Feature Selection by ExtraTreeClassifier

The features extracted by VGG16 are fed to the ExtraTree Classifier, which is a model-based feature selection technique. The Extremely Random Tree Classifier is an ensemble technique that sorts the features with the most votes and generates numerous trees built randomly from the training dataset. The decision trees are fitted to the complete dataset rather than utilizing a bootstrap technique, and the nodes are divided randomly. The splitting is based on randomness, thereby lowering variance. The model’s accuracy will be reduced when irrelevant features are processed and will take more time for computation. The execution time for the classification in this study has been significantly shortened by feature selection employing extra trees classifiers. In ExtraTreeClassifier, the number of trees in the forest can be in the range of 10 to 100, and based on the number of trees, the features selected will be different. We tried other tree-splitting criteria, and the number of features chosen depends on the number of trees in the forest as in Table 1. The highest performance is achieved when the tree splitting criteria is Gini and the trees in the forest are set as 50.

We experimented with different pre-trained models as feature extractors, and the features selected by ExtraTreeClassifier are projected in Table 2. The number of trees in the ExtraTreeClassifier is 50, and the tree splitting criteria is chosen as ‘Gini’.

### 3.3. Classification

The features extracted by VGG16 are selected by utilizing ExtraTreeClassifier and are fed to the Multi-Layer Perceptron (MLP) model as the classifier. The MLP Classifier has an input layer, two hidden layers, and an output layer. The input layer consists of 4323 neurons comprising the features selected by ExtraTreeClassifier. The first and second hidden layers consist of 100 and 70 neurons, respectively, with ReLu activation. The output layer has three neurons with Softmax activation. The VGG16+MLP achieved an accuracy of 99.09%. Accuracy, precision, recall, and F1-Score are standard evaluation metrics that we have used to assess the classification performance. The ratio of accurate predictions to all the input images is called accuracy and is calculated using Equation (4).

(4) $$Accuracy=\frac{TP+TN}{TP+TN+FP+FN}$$

The terms recall, or True Positive Rate (TPR) are also used to describe sensitivity. Equation (5) calculates it, giving an idea of the percentage of positive samples that were accurately predicted as positive.

(5) $$Sensitivity=\frac{TP}{TP+FN}$$

The percentage of all negative samples that were correctly predicted to be negative is what is known as the “True Negative Rate” (TNR) or Specificity and is computed using Equation (6).

(6) $$Specificity=\frac{TN}{TN+FP}$$

Equation (7) can be used to measure precision, which indicates what percentage of positive predictions came true.

(7) $$Precision=\frac{TP}{TP+FP}$$

An evaluation is performed with different adaptive optimizers in the classifier’s performance as in Table 3, and the best accuracy is achieved with the Adam optimizer.

A comparison is performed with other existing pre-trained models as feature extractors, and the results are illustrated in Table 4.

From Table 4, it is evident that the proposed framework ExtRanFS outperformed all other models regarding quality metrics such as accuracy, precision, recall, and F1-Score. Similarly, we have compared with other existing state-of-the-art feature selectors with and without ExtraTreeClassifier as feature selectors.

#### Computational Complexity

An analysis of the mathematical possibilities for effective computer learning is called ‘Computational Complexity of Machine Learning Algorithms’. The total number of trainable parameters accounts for the computational complexity of the model. Table 5 projects the number of trainable parameters and the run time with and without feature selection. With feature selection, the number of trainable parameters and the run time is drastically reduced compared to the run time without feature selection.

**Figure 6:**
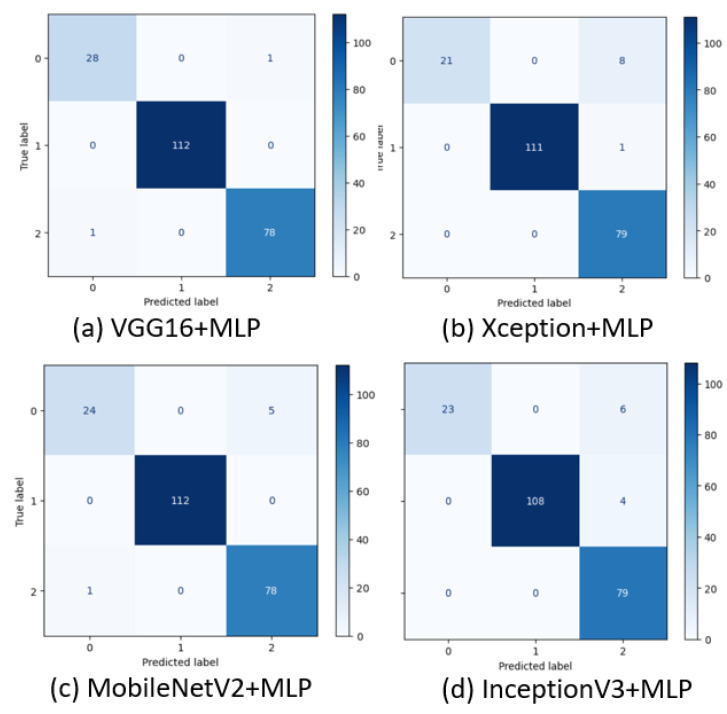
Confusion matrix of the classifier upon feature extraction from different pre-trained models.

When evaluating classification performance, the confusion matrix compares real and predicted values. We obtain a 3 × 3 confusion matrix since there are three classes. The confusion matrix of the classifier upon feature extraction from various pre-trained models is illustrated in Figure 6. Figure 6a represents the confusion matrix of MLP upon feature extraction from VGG16. There is one wrong classification in the benign class and one in the normal case.

Figure 6b illustrates the confusion matrix of MLP upon feature extraction from Xception and has eight wrong predictions in the benign class, along with one wrong prediction in the malignant case. Figure 6c represents the confusion matrix of MLP classifier upon feature extraction from MobileNetV2 and has five wrong predictions in the benign class and one in the normal class. Figure 6d represents the confusion matrix of MLP upon feature extraction from InceptionV3 with six wrong predictions in the benign class and four in the malignant class. From this, it is evident that the MLP classifier with VGG16 as feature extractor outperformed well when compared to other pre-trained models as feature extractors.

**Figure 7:**
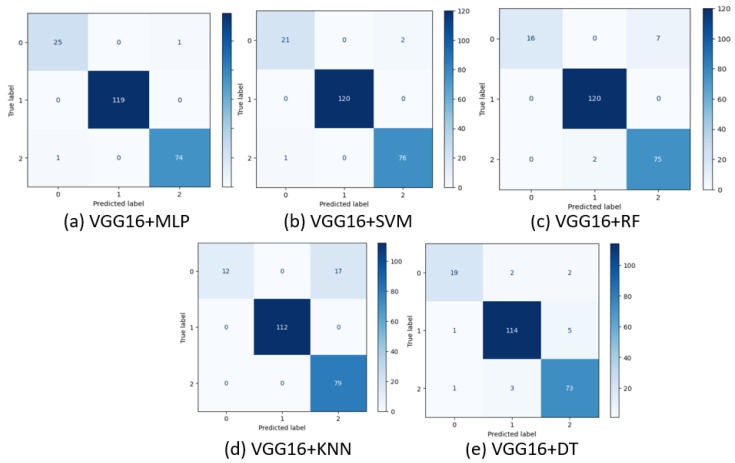
Confusion matrix of various classifiers upon feature extraction from VGG16.

We experimented with different state-of-the-art classifiers upon feature extraction from VGG16. Figure 7a is the confusion matrix of the MLP Classifier with two misclassifications, one in benign and the other in normal cases. Figure 7b has four wrong predictions in the benign category, whereas Figure 7c has a total of eleven misclassifications. Figure 7d shows the confusion matrix of the VGG16+KNN Classifier with fifteen wrong predictions. The Decision Tree Classifier had seven wrong classifications Figure 7e. In this scenario also, the MLP classifier with VGG16 outperformed well.

**Figure 8:**
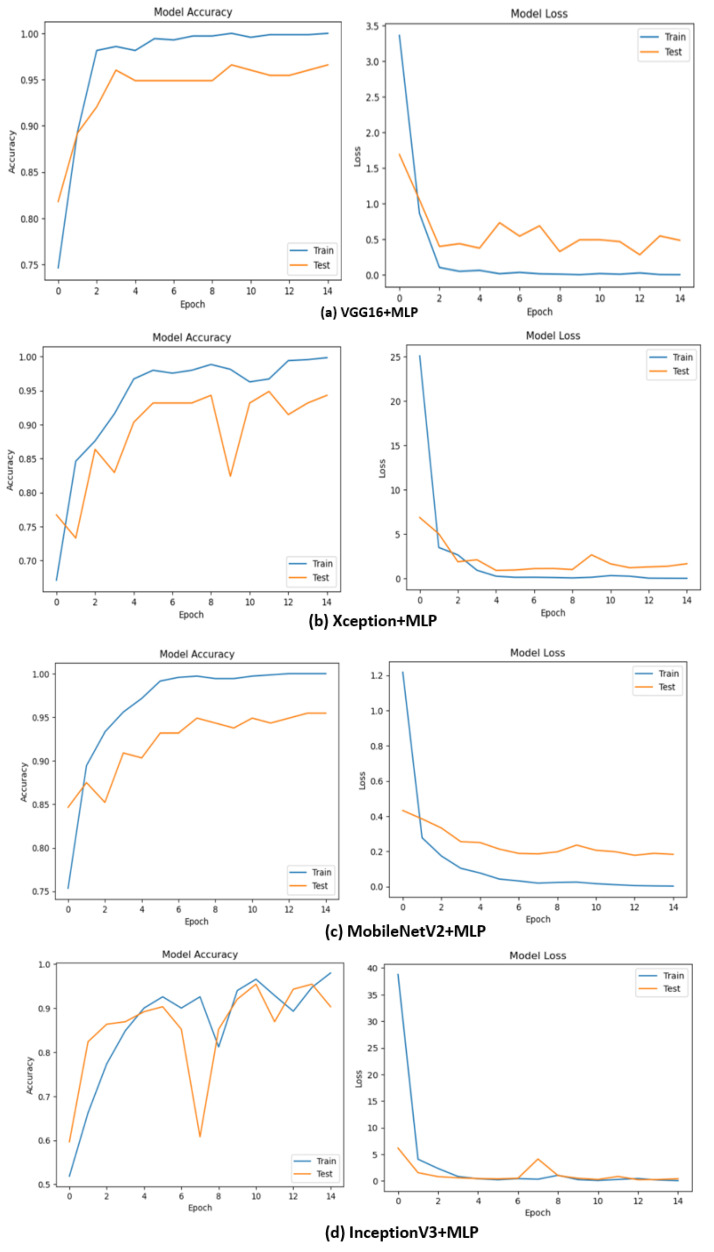
Accuracy and loss of the MLP Classifier upon feature extraction from various pre-trained models.

The accuracy and loss curves are used to evaluate the model’s progress. These are prominently used quality metrices in machine learning. When there is a gap in accuracy between training and testing, overfitting is evident. The overfitting increases as the gap widens. Figure 8 shows the accuracy and loss of the model. The figure shows that the model is not overfitted. According to the ‘Model Accuracy’ graph, training does not require as many epochs as is suggested by the flattening of the curve after the first few rapid rises in accuracy.

Figure 8a shows the accuracy and loss curve of the MLP classifier upon feature extraction from VGG16. From the accuracy curve, it is evident that the model attained around 97.00% accuracy nearly after the second epoch. Since there is no drastic gap between training and testing accuracy, we can say that the model is not overfitted. Figure 8b projects the accuracy loss curves of MLP classifier when pre-trained from Xception for feature extraction. Similarly, Figure 8c illustrates the accuracy loss curve of the MLP classifier upon feature extraction from MobileNetV2. Figure 8d shows the accuracy-loss curve of the MLP classifier on feature extraction from pre-trained InceptionV3.

**Figure 9:**
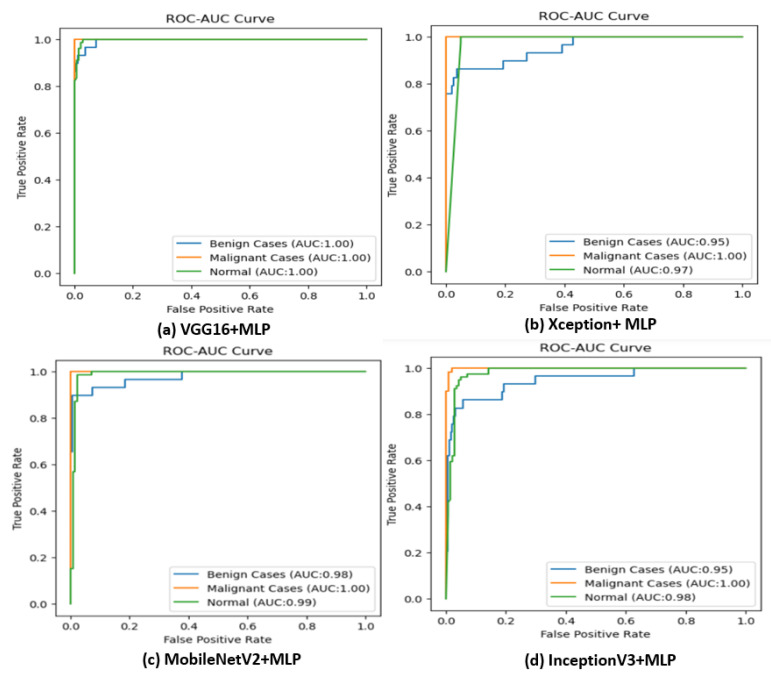
ROC curve of the classifier upon various pre-trained models as feature extractors.

The Receiver Operating Characteristics (ROC) of the classifier is constructed by plotting Sensitivity against Specificity. Classifiers perform better when their curves are closer to the top-left corner. From Figure 9, it is evident that the classifier performed well with different pre-trained models as feature extractors.

## 4. Discussion

Researchers proposed various deep architectures for the detection of lung cancer since deep learning eliminates the necessity of feature extraction. Deep learning works as a black box that performs both feature extraction and classification. Sharaf et al. [16] utilized ExtraTreeClassifier and metaheuristics to classify email. Diana et al. [17] performed leukocyte classification by extracting features using ResNet50, feature selection by ExtraTreeClassifier, followed by SVM Classifier and attained an accuracy of 90.76%. From CT scans, Asuntha et al. [18] identified textural, geometric, and intensity features. Using Convolutional Neural Networks (CNN), these optimized features were employed for categorization and obtained an accuracy of 95.62%. The model could effectively reduce computational complexity. Song et al. [19] experimented with three different deep neural networks for classification and out of which CNN outperformed other models with an accuracy of 84.15%. According to Dutta et al. [20], Convolutional Neural Networks can be used for medical image classification either by training from scratch or by utilizing transfer learning. Sajja et al. [21] used the LIDC dataset for lung cancer detection using GoogleNet and achieved a testing accuracy of 99.00%. In contrast, Rahul et al. [22] used pre-trained models for feature extraction in the survival prediction of adenocarcinoma patients and resulted in 90.00% accuracy.

Gao et al. [23] used pre-trained VGG16 on the LUNA16 dataset for lung nodule detection and obtained an accuracy of 96.86%. In contrast, Chon et al. [24] performed various operations such as segmentation, nodule detection, and classification and achieved an AUC of 0.83. Razeq et al. [25] concentrated on how CNNs classified lung nodules according to the size of the input lung CT, whereas Abdulla et al. [26] performed lung cancer detection using a deep neural network. Chang et al. [27] suggested a two-staged Convolutional Network for lung cancer detection by integrating noise removal. Satti et al. [28] proposed a filter-based noise removal technique for eliminating the commonly occurring impulse noise in medical images. Kaviya et al. [29] conducted a detailed literature review on how artificial intelligence can be effectively utilized in detecting lung cancer.

The authors of [30,31,32,33] used Artificial Neural Networks to classify lung nodules or lung cancer, but the significant limitations were the manual handcrafted feature selection technique. Manual feature selection requires domain expertise, and choosing inappropriate or irrelevant features may result in building a less robust model. The authors of [34] employed LeNet-5 architecture with 10-fold cross-validation and attained a validation accuracy of 97.04%. Wang et al. [35] utilized a transfer learning-based strategy to classify lung cancer, and the model achieved an accuracy of 85.71%. We have evaluated different machine learning models as classifiers, such as Support Vector Machine (SVM), Random Forest (RF), K-Nearest Neighbor (KNN), and Decision Tree (DT), and the results are projected in Table 6. We utilized LinearSVC from sklearn.svm and the loss was calculated using a squared hinge. The one-vs-rest pattern provides the multiclass classification with a penalty selected as l2, and the tolerance for the stopping criterion is set as 1 × 10${}^{-5}$. With 50 trees in the forest, the Random Forest meta-estimator fits a variety of decision tree classifiers to different dataset subsamples. The number of neighbors in the K-Nearest Classifier is set as 7, whereas the tree splitting criteria in DecisionTreeClassifier is set as Gini.

Table 7 compares our proposed framework with other existing state-of-the-art models on the same dataset ‘IQ-OTHNCCD’. The results demonstrate that our proposed classifier obtained the best accuracy of 99.09% when pre-trained with VGG16 as a feature extractor.

AlexNet CNNs were used by Al-Yasriy et al. [36] to identify and categorize lung cancer. The model that was developed had a high accuracy of 93.54%. Nishio et al. [39] tried to classify lung nodules by implementing a transfer-learning-based deep CNN; the authors concluded that larger input images and transfer learning enhanced the Computer Aided Diagnosis(CAD). Humayun et al. [37] incorporated data augmentation on the ‘IQ-OTH/NCCD dataset’, and the classification on pre-trained VGG16 achieved an accuracy of 98.83% for the training data. Still, for testing data, the accuracy was only 86.45% indicating an overfitting model. Kareem et al. [40] utilized the same ‘IQ-OTH/NCCD dataset’ for lung cancer classification by utilizing image enhancement, segmentation, and feature extraction. The features were classified using the SVM classifier, and the authors achieved an accuracy of 89.88%. AL-Huseiny et al. [38] performed various pre-processing involving texture analysis, morphological operation, and ROI Extraction. They used pre-trained GoogleNet architecture for lung cancer classification and obtained an accuracy of 94.38%.

## 5. Conclusions

Transfer learning is the process of using a model that has already been learned to solve a new problem. It is currently highly popular in deep learning since it can train deep neural networks using a minimal amount of data. Transfer learning eliminates the requirement for initial training from scratch. The proposed methodology classified lung cancers into three groups. For the classification challenge, we employed the VGG16 pre-trained model as a feature extractor. The fully connected dense layers of VGG16 are replaced with an MLP Classifier. The proposed classifier outperformed other existing state-of-the-art methodologies with the best accuracy of 99.09%.

## Figures and Tables

**Table 1:** Features selected by different tree splitting criteria.

Trees in Forest	Selected Features (Gini)	Selected Features (Entropy)
10	1285	1013
20	2287	2009
30	3074	2950
40	3722	3690
50	4323	4104
60	4876	4562
70	5225	5143
80	6318	5940
90	6896	6231
100	7200	6921

**Table 2:** Feature extraction and feature selection.

Pre-Trained Model	Features Extracted	Features Selected
VGG16	25,008	4323
Xception	100,352	6140
MobileNetV2	62,720	5191
InceptionV3	51,200	5949

**Table 3:** Comparison of ExtRanFS framework (VGG16+MLP) with various adaptive optimizers.

Optimzer	Accuracy (%)	Precision (%)	Recall (%)	F1 Score (%)
Adam	99.09	98.66	98.66	98.66
Adagrad	94.00	91.66	88.33	89.66
Adadelta	65.00	52.33	51.00	51.66
RMSprop	66.00	71.33	68.33	69.33

**Table 4:** Comparison of ExtRanFS framework with other state-of-the-art methodologies with feature selection.

Classification Model	Accuracy (%)	Precision (%)	Recall (%)	F1 Score (%)
VGG16+MLP (Proposed)	99.09	98.66	98.66	98.66
Xception+MLP	96.00	96.67	90.33	93.00
MobileNetV2+MLP	97.00	96.67	94.00	95.00
InceptionV3+MLP	94.00	90.33	89.66	89.66

**Table 5:** Comparison of ExtRanFS framework with other state-of-the-art classifiers with and without feature selection in terms of run time.

Classification Model	With Feature Selection		Without Feature Selection	
	Trainable Parameters	Run Time (s)	Trainable Parameters	Run Time (s)
VGG16+MLP (Proposed)	445,183	300	2,516,183	660
Xception+MLP	835,583	600	10,042,583	780
MobileNetV2+MLP	762,183	180	6,279,383	300
InceptionV3+MLP	867,483	360	5,127,383	660

**Table 6:** Comparison of ExtRanFS Framework with other state-of-the-art Classifiers.

Classification Model	Accuracy (%)	Precision (%)	Recall (%)	F1 Score (%)
VGG16+MLP (Proposed)	99.09	98.33	98.33	98.33
VGG16+SVM	98.63	96.66	97.33	97.00
VGG16+RF	95.90	89.00	96.33	91.66
VGG16+KNN	93.18	82.33	88.66	84.66
VGG16+DT	93.63	91.00	92.33	91.33

**Table 7:** Comparison of ExtRanFS framework with other state-of-the-art Classifiers.

Author	Architecture	Accuracy (%)	Sensitivity (%)	Specificity (%)
Al-Yasriy et al. [36]	AlexNet	93.54	95.71	95.00
Humayun et al. [37]	VGG16	98.83	Not Specified	Not Specified
AL-Huseiny et al. [38]	GoogLeNet	94.38	95.08	93.70
ExtRanFS (Proposed)	VGG16 +MLP	99.09	98.66	98.00

## Data Availability

https://www.kaggle.com/datasets/waseemnagahhenes/lung-cancer-dataset-iq-othnccd (accessed on 3 January 2023). The source code is available as ‘Automated Lung Cancer Malignancy Detection System—Source Code’ (available since 25 June 2023)—at http://www.mirworks.in/downloads.php.
